# Hypotheses for the Adaptive Maintenance of Phenotypic Polymorphisms

**DOI:** 10.1002/ece3.73493

**Published:** 2026-05-04

**Authors:** Jay J. Falk, Michael S. Webster, Dustin R. Rubenstein

**Affiliations:** ^1^ Department of Neurobiology and Behavior Cornell University Ithaca New York USA; ^2^ Cornell Lab of Ornithology Ithaca New York USA; ^3^ Smithsonian Tropical Research Institute Balboa Ancón Republic of Panama; ^4^ Department of Ecology and Evolutionary Biology Princeton University Princeton New Jersey USA; ^5^ Department of Ecology, Evolution and Environmental Biology Columbia University New York New York USA

**Keywords:** adaptation, balanced polymorphism, balancing selection, female‐limited polymorphism, maintenance selection, phenotypic polymorphism, social selection

## Abstract

Phenotypic polymorphisms have fascinated evolutionary biologists since the field's inception, providing easily observable and quantifiable variation amenable to both empirical and theoretical study. However, identifying the selective mechanisms by which these polymorphisms are maintained can be challenging, in part because alternative hypotheses can be difficult to distinguish. Here we review hypotheses for the maintenance of phenotypic polymorphisms, integrating them into a simple framework in which hypotheses can be described by both of two dimensions: (1) a type of maintenance selection that favors a balanced polymorphism; and (2) a selective context (e.g., ecological, social). Taken together, a phenotypic polymorphism persists through a type of maintenance selection which acts through a selective context. Within each dimension, we provide a path for categorization of alternative hypotheses and their respective predictions. To demonstrate, we explore the case of female‐limited polymorphism, a class of polymorphisms with diverse explanations, yet little unifying theory across taxa. We suggest that, in most cases, negative frequency‐dependence and social competition drive the maintenance of female‐limited polymorphism. Applying this framework to both within‐sex and species‐wide polymorphism reveals distinctions and commonalities across disparate taxa and provides a clear structure for developing and testing hypotheses.

## Graphical abstract

Phenotypic polymorphisms offer easily observable diversity ideal for empirical and theoretical exploration, but developing and comparing hypotheses for the adaptive maintenance of polymorphism can be a challenge. Here, we propose a framework categorizing polymorphisms based on (i) one of five distinct types of maintenance selection and (ii) the context through which selection acts. We use female‐limited polymorphism as a case study, demonstrating key selective drivers of this widespread phenomenon. Applying this framework unveils insights across taxa, guides hypothesis development, and illuminates potential avenues for future research.

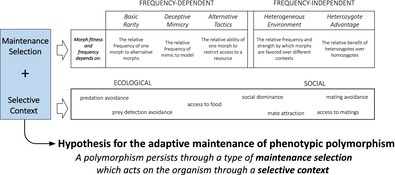

## 1 Introduction

Explaining the maintenance of phenotypic variation in the face of selection is one of evolutionary biology's most enduring challenges (A. R. Wallace 1865; Ford 1945; Levene 1953; Hedrick 2007). Within a population, selection should favor phenotypes with the highest fitness, and drift can also stochastically eliminate variants over time. Yet phenotypic variation is ubiquitous in most organisms. Particularly puzzling are *polymorphisms*, wherein within‐population variation is discrete (Ford 1945, Box 1). Here we emphasize *phenotypic polymorphisms* in which two or more physical “morphs” or “phenotypes” exist in a population. Genetic polymorphisms, in which there is variation at one or more nucleotide sites of a DNA sequence (Box 1), may or may not be linked to phenotypic polymorphisms (Box 2) and are not the focus of this review. Phenotypic polymorphism can also occur within a single sex, and although these sex‐limited polymorphisms are more studied in males (male‐limited polymorphism), they also occur in females (female‐limited polymorphism) across a large number of taxa (Mank 2022). Despite enduring interest in phenotypic polymorphism, determining the selective mechanisms that prevent fixation of a single morph has long proved challenging, in part, because different types of phenotypic polymorphism (e.g., morphological, behavioral) have been studied by different fields, and hypotheses are typically discussed within species, hampering comparison and discussion across taxa and sexes.

BOX 1 **The Definition of Polymorphism.**

For the purposes of this paper, we define *phenotypic polymorphism* as within‐population, discrete, phenotypic variation. However, the definition of polymorphism has changed over time, reflecting shifting frameworks in our understandings of biological variation, and even today has different usages in different fields. The term has long been used to describe morphic variation in and among species (e.g. Darwin 1859). Ford (1940, 1945) provided a more concrete, phenotypic, within‐population definition: “the occurrence together in the same habitat of two or more distinct forms of a species in such proportions that the rarest of them cannot be maintained by recurrent mutation”. Later frameworks (Michener 1961; Mayr 1963) focused on heritability, splitting terminology such that *polymorphism* referred to morphs associated with genetic differences while *polyphenism* (synonymous with “conditional” tactics) referred to variants that are environmentally determined.

The dichotomy between polymorphism and polyphenism, which is based largely on trait development, has been highly influential and is an important framework for understanding and categorizing discrete traits (Gross 1996; West‐Eberhard 2003b; Oliveira et al. 2008; Mank 2022). While developmental categorization of discrete phenotypes as either polymorphism or polyphenism has value in many contexts, there are also drawbacks. First, it can be difficult to know the heritability of a trait of interest during the initial phases of research (i.e., those with long generation times, that are difficult to observe in the wild, etc.). Second, all phenotypic variation reflects both genetic and environmental effects (Huxley 1942), and dividing terminology excludes cases of intermediate heritability and gene‐by‐environment interactions (e.g. Geffroy et al. 2021). Third, some usages of the term polyphenism only include discrete variation, whereas others include all forms of environmentally determined variation regardless of trait distribution (Canfield and Greene 2009). Reality is more complex than a simple dichotomy of genetic polymorphism versus environmental polyphenisms, overlooking critical details—such as the fact that environmental sensitivity can vary within populations and evolve over time (Lively 1986; Plaistow et al. 2004).

We therefore suggest the re‐adoption of Ford (1940, 1945) usage of the term polymorphism in all cases of discrete variation, regardless of trait heritability. When necessary, polymorphisms can be referred to as heritable or nonheritable, with polyphenism referring to a less heritable polymorphism. We use this generalized definition of *phenotypic* polymorphism to describe all discrete phenotypic variation. It is also important to note that in the field of population genetics, polymorphism has a specific definition of variation in a DNA sequence, regardless of its effect on phenotype. We therefore refer to “phenotypic polymorphisms” in this review to distinguish our meaning and to ensure a clear distinction from the term's usage in population genetics.

BOX 2 **Maintenance Selection Versus Balancing Selection.**

Balancing selection is typically used to describe selective processes that favor persistence of multiple alleles at a locus. However, in this paper we are describing selection that favors persistence of discrete phenotypes, not necessarily genotypes. Therefore, we use the term “maintenance selection” rather than balancing selection because balancing selection in a strict sense may or may not be at play. In each type of maintenance selection that we describe, selection acts on the phenotype in a way that promotes persistence of the polymorphism, regardless of underlying genetic architecture (heterozygote advantage is the only mechanism that is inherently genetic). Maintenance selection is distinct from diversifying or disruptive selection because it involves multiple phenotype persistence, not necessarily divergence.

Morphic variation can arise from highly varied genetic architectures and modes of selection. In one scenario, a single genetic locus could determine the presence or absence of a phenotypic polymorphism, with each allele associated with each phenotypic morph. This is a classic “genetically controlled” polymorphism where genetic polymorphism and phenotypic polymorphism are aligned. In this case,  the phenotypic polymorphism persists through balancing selection on the genetic polymorphism.

On the other hand, an individual's development into one morph or another could be determined by external environmental factors like diet or temperature (i.e., plastic, conditional, environment‐dependent, polyphenic traits). While it is the environment that determines the morph, reaction norms—including slopes and switchpoints—are often heritable and can evolve. For example, many turtles determine sex based on external temperatures during embryonic development, but families vary in the threshold temperature by which an individual becomes male or female, implying a heritable component to the threshold temperature (Bull et al. 1982; Kocher et al. 2024). In spadefoot toads, a diet polyphenism is triggered by tadpole pond environment, yet innate propensity to develop into one type or another, and their degree of plasticity are both genetically variable (Levis et al. 2018; Isdaner et al. 2024). Though developmentally plastic, in both of these cases, there can still be adaptive selection for the maintenance of the phenotypic polymorphism, because environmental thresholds are likely to be polygenic and contain some degree of heritable variation within a population (West‐Eberhard 2003b). Maintenance selection refers to selection that maintains phenotypic morphs, rather than allelic variation. Therefore, it may include balancing selection on alleles, but does not require it.

Paradoxically, this implies that maintenance of phenotypic polymorphism does not necessarily require the maintenance of genetic diversity. Stabilizing selection could maintain a narrow switchpoint in the population, or directional selection could cause a shift in the switchpoint that leads to more or less phenotypic variation. A scenario can also be envisioned in which a locus has two alleles with distinct reaction norms, resulting in discrete phenotypic morphs in the same environment. In this case, balancing selection could play a role to maintain both versions. It may be tempting to distinguish between selection types for either genetic‐ or environmentally‐based polymorphisms, but as we discuss in Box 1, most variants are likely to contain elements of both, and some polymorphisms are polygenically determined, complicating a single allele – single morph model of a genetic polymorphism. The terminology of maintenance selection allows us to discuss selection on phenotypes without making assumptions of genetic or developmental underpinnings.

One such division is between the study of discrete variation that is determined by genetic variation versus environmental conditions (i.e., polymorphism versus polyphenism, see Box 1). Although sometimes described as distinct, these phenomena are in fact two ends of a spectrum that depend on levels of developmental plasticity (see Box 1 and Box 2). Natural selection acts on phenotypic variation regardless of the developmental or genetic mechanisms by which that variation arises. Whether a trait is primarily environmentally induced or genetically determined, selection operates on its expressed form and its consequences for fitness, not on the underlying causal pathway (Lande and Arnold 1983; West‐Eberhard 2003b; Svensson 2023). It follows that overreliance on such development‐based frameworks might limit the construction and comparison of alternative adaptive hypotheses for the maintenance of polymorphism (we use the term *adaptive* to mean the selective pressures that maintain the presence of multiple phenotypes, not the developmental, genetic, or evolutionary origins of the phenotype). For example, Gross's (1996) classic review of male‐limited polymorphism is divided into two sections—evolutionary studies and proximate studies. Hypotheses for the “evolution” of male polymorphism were classified as either alternative, mixed, or conditional strategies. Yet, these categorizations are based on differences in the developmental origin of morphs, not what maintains the polymorphism. Certainly, the consequences of selection depend a great deal on heritability, but this should not be confused with the causative type of selection that maintains variation. Condition‐dependence and negative frequency‐ dependence are not alternative explanations because condition‐dependent traits can be balanced under negative frequency‐dependent selection (Hazel et al. 1990; Shuster and Wade 2003; Tomkins and Hazel 2007, Box 2). We should instead consider condition dependence as a proximate mechanism for the development of multiple morphs, but it cannot alone explain the adaptive maintenance and persistence of such morphs. Therefore, while frameworks that primarily distinguish between genetically and environmentally determined phenotypes are useful in many contexts, they are not appropriate for developing clear alternative hypothesis for the adaptive maintenance of multiple phenotypic morphs.

When testing explanations for the maintenance of a phenotypic polymorphism, alternative hypotheses are often unconsidered or untested. For example, the hypothesis that male‐limited polymorphism is often related to alternative reproductive tactics for access to mates has ample support (Gross 1996; Oliveira et al. 2008; Mank 2022), yet alternatives to this hypothesis are rarely tested or even considered (e.g., competition for access to food). In another example of untested hypotheses, the term “mimicry” is often used loosely, and sometimes only based on resemblance to females by human standards (Jukema and Piersma 2006). As we discuss below, mimicry and alternative reproductive tactics are in fact two independent ways in which maintenance selection can maintain the coexistence of multiple morphs. Both involve negative frequency‐dependence, but the theoretical parameters that govern stable levels of each morph are distinct. It is certainly possible—if not likely—that these two mechanisms can interact in tandem. However, when female mimicry is hypothesized to be part of an alternative tactic, critical aspects of mimicry often remain untested, such as the encounter rates between alternative male morphs and females, and whether this alters mimic effectiveness (e.g., Xu and Fincke 2011 with male mimics). Failure to verify and test these alternatives can lead to inaccurate narratives and long‐term misinterpretations (Kunte 2009; Kamath and Wesner 2020).

**FIGURE 1:**
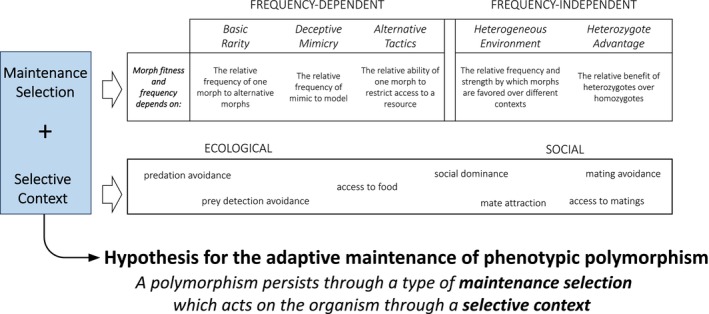
A simple method for developing hypotheses for the adaptive maintenance of phenotypic polymorphism. An adaptive hypothesis should involve at least one type of *maintenance selection* (either frequency‐dependent or frequency‐independent) and at least one selective *context* through which maintenance selection acts (ecological and/or social). Each type of maintenance selection is theoretically capable of maintaining a polymorphism, but actual polymorphisms may involve multiple types of maintenance selection and selective contexts.

Here, we review hypotheses and outline a simple conceptual framework for developing and distinguishing hypotheses for the adaptive maintenance of phenotypic polymorphism (Figure 1). We propose that a hypothesis for the maintenance of phenotypic polymorphism should be described by two distinct dimensions: (1) a type of maintenance selection—the selective process that maintains multiple morphs within a population; and (2) a selective context—the aspect of the organism's life history upon which the maintenance selection acts (e.g., competition for food or for mates—ecological and social context). Both factors are important for describing alternative hypotheses for the maintenance of phenotypic polymorphism and should be thought of as separate and independent dimensions of a hypothesis. Just as directional selection can function under many contexts (e.g., ecological, social, sexual, or sexual conflict), so too can selection for polymorphisms, and defining this context is critical to understanding and testing alternatives.

This framework pertains to explanations for how phenotypic polymorphisms are maintained rather than the evolutionary origins of novel morphs or the developmental bases of such morphs. We do not address the process by which discrete variants arise, nor how they remain discrete through forces such as disruptive selection (Kitano et al. 2025). Instead, we ask why multiple discrete morphs are maintained rather than a single morph favored. Although we see no particular reason why this framework should be limited to animals, we mostly use examples from animal taxa. Our purpose is not to assign cases of phenotypic polymorphism to rigid categories, but instead to define, refine, and clarify major ways in which hypotheses for such polymorphism can be described. Ultimately, our hope is to provide a process for generating testable hypotheses during the study of a phenotypic polymorphism. We imagine a scenario where researchers encounter a phenotypic polymorphism in nature and wish to test alternative hypotheses for how it is maintained. As such, we provide concrete predictions that can be used to test and distinguish these hypotheses in the field (Figure 1).

In the last section, we use our framework to categorize instances of female‐limited polymorphism, a class of phenotypic polymorphisms with diverse and varied explanations (Svensson et al. 2025). We highlight similarities and differences between male‐ and female‐limited polymorphism, and demonstrate that female‐limited polymorphism, much like female ornamentation, is driven by strong social competition for resources other than mates. Both maintenance selection and selective context have long been aspects of a vibrant discussion on polymorphism. Formalizing the distinction between these descriptive dimensions helps to clarify similarities and differences in phenotypic polymorphism across animal taxa, allows for simple construction of testable alternative hypotheses, and reveals the many ways in which selection maintains phenotypic diversity.

## 2 Why Two Dimensions?

To begin, we provide two examples of phenotypic polymorphism in nature to demonstrate the development of alternative hypotheses. We detail all categorizations in the following sections. In brief, maintenance selection includes three types of negative frequency‐dependent selection—basic rarity, deceptive mimicry, or alternative tactics; or frequency‐independent selection—heterogeneous environments or heterozygote advantage. The selective context is the aspect of an organism's natural history that is hypothesized to be under maintenance selection. Each hypothesis describes polymorphisms as being maintained by one or more types of maintenance selection, which act on the organism through one or more selective contexts (Figure 1). Describing both dimensions clarifies the testable predictions of each hypothesis and creates a framework for easy comparison across taxa.

In both damselflies and hummingbirds, female‐limited polymorphism has evolved multiple times. Females of many damselfly species have two or three morphs—one which appears like the male in coloration (androchrome), while the others do not (heterochromes or gynochromes) (Verhaar 1985; Askew 2004; Willink et al. 2025). In this system, it may benefit females to avoid excessive matings because copulation can take hours, unnecessarily exposing them to predators and taking time from feeding and laying eggs (Robertson 1985). Androchrome females might deceive males by mimicking them, thereby evading male attention more than heterochromes (Robertson 1985). The effectiveness of this strategy, however, is dependent on androchrome females being rare relative to males—as mimicry increases, its fitness also decreases, perhaps resulting in a balanced polymorphism. When developing a hypothesis, the type of maintenance selection (dimension 1, Figure 1) informs us of the relevant players and dynamics between them, whereas the selective context (dimension 2, Figure 1) informs us of the life history stage or behavior where we are likely to see these dynamics. Therefore, in this hypothesis, *deceptive mimicry* is what maintains the polymorphism, and *mating avoidance* is the selective context in which mimicry acts. Many forms of this hypothesis have been proposed for polymorphisms in damselflies (reviewed in Fincke 2004; Van Gossum et al. 2008). An alternative is that male damselflies learn to recognize potential female mates, and female polymorphism makes it difficult for males to cue in on *any* particular female morph (Miller and Fincke 1999; Fincke 2004). In this case the type of maintenance selection would be *basic rarity* advantage rather than mimicry, but *mating avoidance* is still the context. Between these two hypotheses, dimension 1 differs, but dimension 2 remains the same. Although both of these mechanisms—*deceptive mimicry* and *basic rarity*—involve negative frequency‐dependence, the prediction of each hypothesis is distinct: under deceptive mimicry, the male to male‐mimic ratio governs the balancing dynamics of the system, whereas under a basic rarity hypothesis, the male to male‐mimic ratio is irrelevant, and it is the ratio between all female morphs that matters for the strength of selection and equilibrium frequencies of each morph. We note also that under basic rarity, the similarity to males is irrelevant to polymorphism maintenance, while under deceptive mimicry, it is crucial.

Like damselflies, white‐necked jacobins ( *Florisuga mellivora* ) and some other hummingbird species can either be androchromic or heterochromic (Diamant et al. 2021; Falk et al. 2021). Rather than involving the context of *mating avoidance*, androchrome female white‐necked jacobins appear to be *avoiding aggression* from other hummingbirds around food resources. Appearing like the more aggressive male white‐necked jacobins gives androchromic females access to more nectar resources (Falk et al. 2021, 2022). Like the dimension 1 hypothesis in damselflies, maintenance selection in these hummingbirds involves *deceptive mimicry* of males, but here it is dimension 2, the selective context, that differs from that in damselflies. *Mating avoidance* is the context for male mimicry in damselflies, whereas in female hummingbirds, males are mimicked to *avoid aggression* and to gain access to food resources. Thus, the type of maintenance selection, deceptive mimicry, shares similarity to the first damselfly hypothesis described above, but in white‐necked jacobins it is not clear that females benefit from mating avoidance. Instead, it is the selective context that differs—appearing like a male has demonstratable benefits to avoiding aggression at food resources (Falk et al. 2021). Damselflies and hummingbirds demonstrate how a framework that includes a type of maintenance selection and a selective context allows for simple and intuitive comparisons between similar phenomena in different taxa. Next, we detail these concepts and the major forms of each dimension, maintenance selection and selective context.

## 3 Two Descriptive Dimensions of Phenotypic Polymorphism Maintenance

### 3.1 Dimension 1: Maintenance Selection and the Adaptive Persistence of Phenotypic Polymorphism

**FIGURE 2:**
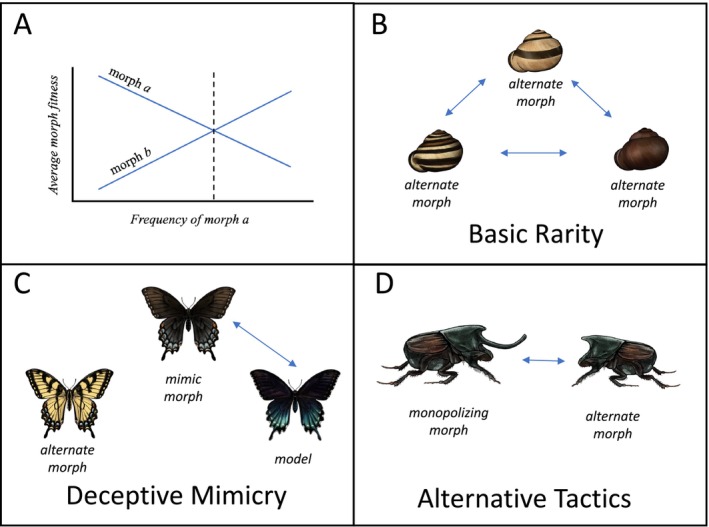
Negative frequency‐dependent selection: (A) A graphical depiction of negative frequency‐dependent fitness which will favor a polymorphism of two morphs, *a* and *b*. The relative fitness of *a* over *b* decreases as its frequency in the population increases. Since *a* has higher fitness when at lower frequencies, its prevalence in the population should increase until it reaches an equilibrium frequency (dotted line). At frequencies greater than equilibrium, *b* outcompetes *a*. A stable polymorphism with equilibrium can result so long as fitness curves for *a* and *b* intersect such that *a* has greater relative fitness at lower frequencies of *a*, and *b* has greater relative fitness at higher frequencies of *a*. One example is depicted here, but other fitness curves are possible so long as this condition is met. (B–D) Representative species of the three forms of negative frequency‐dependent selection. Arrows indicate interaction pairs that influence morph frequency and fitness. (B) Polymorphism in grove snail ( *Cepaea nemoralis* ) shell patterning has been suggested to arise from a variety of selective pressures, including rarity advantage (Clarke 1962). (C) Female eastern tiger swallowtails (*Pailio glaucus*) are polymorphic. One morph (left) has wing coloration similar to males, while the other (right) mimics a sympatric species which sequesters toxic compounds (Kunte 2009). (D) Male dung beetles ( *Onthophagus nigriventris* ) have male‐limited polymorphism in horn size that represents alternative tactics for accessing mating opportunities (Simmons and Emlen 2006). Illustrations by Liz Wahid.

Unlike directional selection that favors fixation of alleles and phenotypes, balancing selection can favor the maintenance of multiple alleles within a population and thus the coexistence of multiple morphs. However, balancing selection is typically defined as selection that maintains multiple alleles, without regard to phenotype. As we discuss in Box 2, selection for the maintenance of balanced polymorphisms does not necessarily require balancing selection of multiple genetic alleles, and balancing selection does not necessarily result in multiple phenotypes. We use the term *maintenance selection* to specifically refer to selection on phenotypes, regardless of the underlying genomic architecture of the trait. There is overlap in the concepts of maintenance and balancing selection because mechanisms that select for multiple alleles can also select for multiple phenotypes. Three types of balancing selection are generally recognized: (1) negative frequency‐dependence; (2) heterogeneous environments; and (3) heterozygote advantage (Hedrick 2007). These types of selection can also be used to discuss selection on phenotypes and are therefore also forms of maintenance selection. Most forms of negative frequency‐dependent selection—when the fitness benefit to a phenotypic morph decreases as its prevalence increases (Figure 2A)—fall under the distinct categories of: (1) basic rarity, (2) deceptive mimicry, and (3) alternative tactics (Figure 1). Our descriptions of these individual mechanisms are not novel, but this delineation of negative frequency‐dependent selection simplifies and clarifies the important variables by which each is governed, allowing for more targeted predictions (Figure 1). The other two mechanisms of maintenance selection, heterogeneous environments and heterozygote advantage, are frequency‐independent, meaning that the average fitness of a phenotypic morph or genotype does not depend on its frequency in the population. Our reference to frequency‐independent and frequency‐dependent does not include frequency‐dependence of allelic “marginal” fitness (Ruzicka et al. 2025), nor the strict usage associated with soft selection (*sensu* B. Wallace 1975). The types of maintenance selection we describe can structure phenotypic dynamics even when morph expression is environmentally induced and does not entail the maintenance of underlying allelic polymorphism (except heterozygote advantage, which is an explicitly genetic concept). In such cases, selection may operate at the phenotypic level without constituting balancing selection in the strict population‐genetic sense (see Box 2 for more discussion).

We emphasize these five categories of maintenance selection as distinct because each is theoretically sufficient to maintain a phenotypic polymorphism on its own, and each is associated with distinct, testable, predictions at the organismal level (Figure 1). However, individual examples of phenotypic polymorphism need not fall neatly into a single category, and many likely result from multiple mechanisms (see Section 3.1.4).

#### 3.1.1 Three Forms of Negative Frequency‐Dependent Selection

##### 3.1.1.1 Basic Rarity

Selection may directly favor rare phenotypes (Fisher 1930) for no reason other than that they are rare. These cases are frequency‐dependent since rare phenotypes will increase in frequency until they are no longer rare, at which point rarer morphs are favored. Basic rarity advantages typically emerge from biases in the behavior of interacting organisms—either a preference or attraction for rare morphs or an avoidance of common ones. For example, basic rarity advantage may appear if predators develop search images for common prey and rare prey morphs benefit by not resembling those types (Endler 1978; Bond 2007), or when signal receivers prefer rare phenotypes, such as female mate preference for novel male phenotypes (Hughes et al. 2013).

All forms of negative frequency‐dependent selection benefit rare phenotypes, but unlike the other forms discussed below, the fitness advantage of one morph over others rests on its relative frequency to other morphs (Figure 2B). In other words, basic rarity does not require any cost/benefit tradeoffs (Box 3) inherent to any particular morph. External factors can affect the precise equilibrium point, including the cognitive or sensory biases of direct competitors or mediators of selection (e.g., predators, prey, mates, etc.) (Fincke 2004), but relative rarity to other morphs is still the factor that drives morph maintenance.

BOX 3 **The Role of Trade‐Offs in the Maintenance of Polymorphisms.**

Different phenotypes often vary in their costs and benefits, creating tradeoffs. The role of tradeoffs in polymorphisms maintenance, however, must be considered carefully. Without a type of maintenance selection, net costs and benefits of each morph will almost always be non‐zero, favoring one rather than multiple phenotypes. When tradeoffs (sometimes referred to sexual, niche, or life history “antagonisms”) are invoked, another type of maintenance selection that interacts with these costs and benefits is typically necessary. For example, tradeoffs often inherently imply a heterogeneous environment or fluctuating conditions, such that neither morph is consistently at an advantage over others (Levene 1953). In these cases, it is the heterogeneous environment's interaction with the tradeoff that leads to maintenance, not the tradeoff alone.

Tradeoffs can also play important roles in other forms of maintenance selection. For example, in a game‐theoretic model of male mimicry by females in white‐necked jacobin hummingbirds, females gain a feeding advantage by mimicking males, but incur a cost when nesting due to their ornamented plumage which draws attention from predators (Falk et al. 2025). The cost of nesting with ornamented plumage is a critical component that dictates the likelihood of mimicry to occur, but it is constant regardless of morph frequencies. Therefore, while this tradeoff plays an important role, it is the negative frequency‐dependence of the mimicry strategy that ultimately drives polymorphism maintenance, not the cost of ornamentation at the nest.

Intralocus sexual conflict, or sexual antagonism, is a type of tradeoff where a morph‐associated trait can be beneficial to one sex but not the other, preventing the trait from loss or fixation (Rice 1992; Connallon and Clark 2014). Such sexually antagonistic selection is conceptually and mathematically similar to selection across heterogeneous environments, as the fitness consequence of a trait depends on the genomic environment, in this case the sex, of the organism expressing it (Kasimatis et al. 2017). This includes genetic interactions, but also differences in natural history and in the factors that increase and decrease male versus female lifetime fitness. One example of sexual antagonism is in the ruffs, where the allele that leads to the female‐similar “faeder” morph has a deleterious effect when carried by females, but male carriers of the faeder morph tend to have higher fertilization success (Giraldo‐Deck et al. 2022). Antagonistic pleiotropy, in which there is a life‐stage tradeoff, has also been hypothesized as a mechanism of balancing selection, but requires heterozygote advantage in order to balance a polymorphism, a condition not required by sexually antagonistic selection (Hedrick 1999; Ruzicka et al. 2025).

Overall, trade‐offs play a central role in shaping the fitness landscape of polymorphic traits (but are also not required for maintenance selection to occur, see section on Basic Rarity). They rarely act in isolation, but rather interact with environmental, genetic, or frequency‐dependent mechanisms to stabilize polymorphisms. Though differential costs and benefits across morphs, trade‐offs influence the conditions under which maintenance selection can operate, but it is unlikely that they can act alone.

As an example, Fisher's early theory for the maintenance of a balanced sex ratio exemplifies rarity advantage (Fisher 1930). Under a skewed ratio for either sex, the average fitness of the rare sex will be higher, assuming a diploid, non‐hermaphroditic species (Fisher 1930; Conover and Van Voorhees 1990). Individuals that produce more of the rare sex will have increased fitness. Importantly, no other properties of females or males per se are necessary to explain the maintenance of a balanced sex ratio—it is simply the fitness advantage of the rare sex. In the same vein, we also expect to see basic rarity advantage in any form of disassortative mating between distinct morphs. For example, in white‐throated sparrows ( *Zonotrichia albicollis* ), white‐stripe and tan‐stripe morphs tend to mate with each other resulting in balanced offspring ratios of these two morphs (Hedrick et al. 2018). Apostatic selection, in which a predator develops a search image for common prey forms and rarer forms resist detection, is another well‐studied example of how this type of basic rarity advantage might manifest (Clarke 1962; Bond 2007). For example, adder snakes ( *Vipera berus* ) can be either patterned or melanistic, and the maintenance of these morphs is likely due to increased predation from crows on more common morphs (Madsen et al. 2022).

##### 3.1.1.2 Deceptive Mimicry

Deceptive mimicry occurs when one group of animals (mimics) appear similar to another group (models) such that a mimic is misinterpreted to be a model, resulting in a benefit to the mimic (Fisher 1930) (Figure 2C). The effectiveness of mimicry decreases as the relative frequency of mimics to models increases (i.e., negative frequency‐dependence), potentially leading to a balanced polymorphism. Batesian mimicry refers to a form of deceptive mimicry where the model is toxic or distasteful to a predator, but the mimic is not. In this case, selection is imposed by a predator that learns to avoid the prey with the model's appearance (Bates 1862; Fisher 1930). However, similar principles may apply in other scenarios when one class of individuals deceptively mimics another, such as when one species mimics a socially dominant species to gain access to food resources (Rainey and Grether 2007; Prum 2014; Miller et al. 2019; Falk et al. 2021). Although in many cases all individuals of a species or population mimic another sex or species, this need not be the case, and mimicry can manifest as a polymorphism if mean mimic and non‐mimic fitness reach equilibrium before mimic frequency reaches fixation (Clarke 1964; Barrett 1976; Kunte 2009; Shine et al. 2022; Falk et al. 2025).

All types of negative frequency‐dependent selection favor rarity, but deceptive mimicry differs in a critical point: in deceptive mimicry the average fitness of a morph depends on the relative frequency of the mimic to the model, not the frequency of one morph to another morph. The frequency of mimics may also depend on several factors including the degree of model‐mimic resemblance, the rate of encounter with the model, and the cost of mistaking a model for a mimic or vice versa (Fisher 1930; Pfennig et al. 2001).

Deceptive mimicry has been experimentally modeled, such as Brower's (1960) classic demonstration using the European starling ( *Sturnus vulgaris* ) as a predator and mealworms as either palatable or unpalatable prey (dipped in quinine). When mimic frequency was low almost all mimics survived, but as their frequency rose their survival rate decreased. In non‐venomous turtle‐headed sea snakes ( *Emydocephalus annulatus* ), banded individuals mimic several species of venomous snakes, and this morph fluctuates with a black morph as expected under negative frequency‐dependence (Shine et al. 2022). Similar principles will apply in any deceptive mimicry system (Jamie 2017). For example, male bluegill sunfish ( *Lepomis macrochirus* ) sometimes mimic females to access egg fertilizations at the nests of non‐mimic males (Dominey 1980). In this case mimics advertise a benefit rather than a danger to the receiver, a non‐mimic male (Jamie 2017).

##### 3.1.1.3 Alternative Tactics

Alternative *reproductive* tactics are discrete reproductive behaviors or phenotypes that occur within a sex (Gross 1996; Oliveira et al. 2008; Kustra and Alonzo 2025). Here, we use the generalized term “alternative tactics” to broaden context beyond within‐sex tactics to include non‐reproductive tactics that may or may not be restricted to a single sex. In the case of a phenotypic polymorphism, one morph uses a tactic to monopolize a resource, while another increases its fitness by exploiting or competing against the monopolizing tactic with a different tactic. Morphological associations with behavioral tactics may facilitate these differences in behavior, such as enlarged weaponry in monopolizing morphs or color signals that display behavioral type (Figure 2D).

The stability and negative frequency‐dependence of alternative tactics have been explored extensively (reviewed in Shuster 2010). In short, high frequencies of the monopolizing tactic create conditions in which some individuals exclude other individuals from access to a critical resource or social interaction like matings or food. Alternative tactics that do not engage in the monopolizing tactic are successful at low frequency, but as their numbers increase, their average success decreases. A phenotypic polymorphism that results from this dynamic is a type of Evolutionary Stable Strategy (i.e., ESS, Smith 1982). The resulting stable frequencies of each morph that are maintained by alternative tactics include the relative ability of the monopolizing morph to restrict access from other individuals, as well as the rate and degree of success of the alternative tactics in competing against the monopolizing morph (Shuster and Wade 2003; Shuster 2010).

A classic example of alternative tactics is the three alternative morphs of marine isopods ( *Paracerceis sculpta* ). Females associate and live inside sponges, and α‐males compete for exclusive access to these female aggregations. Female‐resembling β‐males and small γ‐males use non‐exclusionary behaviors to access females that are guarded by α‐males (Shuster 1992). As aggregations increase in size, β‐ and γ‐males are able to escape detection by α‐males and mate more often, resulting in equal lifetime fitness of each male morph (Shuster and Wade 1991).

#### 3.1.2 Two Forms of Frequency‐Independent Selection

##### 3.1.2.1 Heterogeneous Environments

Fluctuating conditions over space, time, or other contexts (see Box 3) may favor the existence of multiple morphs through specialization for certain conditions (Hedrick et al. 1976; Hedrick 1986). For example, a morph may exist in relatively low frequencies in most years, but under certain environmental conditions it may be favored. Fluctuating conditions may be either biotic (e.g., predation pressure) or abiotic (e.g., climatic variation) and include changes in population density of conspecifics. Under heterogeneous environments, the frequency of morphs depends on the frequency of differing habitats, contexts, or conditions, and each morph's degree of specialization to their respective environment.

For example, an annual‐perennial polymorphism is widely distributed across the yellow monkey flower ( *Mimulus guttatus* ) range and contributes to local adaptation to dry versus wet environments despite gene flow (Twyford and Friedman 2015). In Asian burying beetles (*Nicrophorus negalensis*), a behavioral polymorphism in which beetles either breed year‐round or seasonally is maintained by temperature‐related variation in interspecific competition for resources (Tsai et al. 2020). At high elevation where conditions are cooler and blowflies are generally less abundant, beetles breed year‐round, whereas at low elevation where conditions are warmer, beetles only breed in winter when blowflies are less abundant. *Colias* butterflies are another curious example, where females are either alba (white) or yellow, and alba females have substantially higher body fat, allowing for larger eggs (Graham et al. 1980). One study has shown that alba morphs may be harassed by heterospecific male pierine butterflies when their densities are high, and it is variation in pierine density across their range that may prevent fixation of either morph (Nielsen and Watt 2000). Here it is the biotic environment created by heterospecifics that fluctuates over time. Lastly, we note that sexual antagonisms (and other such trade‐offs or “antagonisms”) are conceptually and mathematically similar to heterogeneous environments, and can also contribute to maintenance selection, which we discuss in Box 3.

Polymorphisms induced through phenotypic plasticity during development in heterogeneous environments are numerous (Nijhout 2003; West‐Eberhard 2003a). For instance, larval tiger salamanders ( *Ambystoma tigrinum* ) can develop into a cannibalistic morph that feeds mostly on other salamanders, swims faster, and has a larger head and teeth than the non‐cannibalistic morph, which feeds mostly on insects (Collins and Holomuzki 1984). The morph is plastically induced through increased tactile stimulation from conspecifics when densities are high (Hoffman and Pfennig 1999). We note that in these plastic polymorphisms, the environmental triggers that cause different morphs in the proximate sense may or may not be the same selective context that leads to adaptive maintenance of the morphs (Moran 1992).

##### 3.1.2.2 Heterozygote Advantage

A heritable polymorphism can be maintained when there is genetic overdominance (also referred to as heterozygote advantage). In overdominance, individual fitness is higher for heterozygotes compared to homozygotes at a genetic locus (Fisher 1923; Dobzhansky 1955). In this type of selection, the stable phenotype frequency depends on the relative fitness advantage of heterozygotes over homozygotes (Fisher 1923).

Although heterozygote advantage is a common aspect of phenotypic polymorphisms, there are few examples in which it unambiguously maintains a phenotypic polymorphism with no influence from other balancing mechanisms. For example, overdominance and disassortative mating (see above, Basic Rarity) are often found together because overdominance creates conditions that favor the evolution of disassortative mating through reduced fitness of homotypic matings. In one example of overdominance that does not apparently involve disassortative mating, Neotropical tortoise beetles ( *Chelymorpha alternans* ) are polymorphic with five different color morphs related to variation at a single gene (Strickland et al. 2019). Captive beetles mate randomly, but clutch hatching and pupal eclosion rates were both lower between parents of the same morph (Strickland et al. 2021). Heterozygote advantage may play a role in maintaining this polymorphism, but the reason for reduced survival rates is currently unknown.

#### 3.1.3 Non‐Adaptive Null

An alternative to these adaptive hypotheses is that the phenotypic polymorphism is not actively maintained by selection, but instead by factors such as mutation‐selection balance or relaxed selection (Kimura 1979). While possible, a neutral or non‐adaptive polymorphism should be unstable in the presence of drift. Some studies explicitly examine morph frequencies under null models to test the explanatory power of drift (e.g., Madsen et al. 2022). This hypothesis should also be considered if adaptive hypotheses are refuted. Our focus here is on the adaptive maintenance of within‐population polymorphism, so we do not discuss hypotheses involving migratory gene flow and hybridization, even though this has been proposed and is supported in some studies (e.g., Cooper 2010; Cooper et al. 2016). However, we note that this type of non‐adaptive evolution may exist on a spectrum with heterogeneous patchy environments described above (e.g., Rosenblum 2006).

#### 3.1.4 Multiple Truths: The Rule Rather Than the Exception

The five types of maintenance selection that we describe above are, under the appropriate conditions, capable of maintaining a phenotypic polymorphism on their own. However, evidence for multiple types of maintenance selection acting on the same polymorphism is common (see Table 1), and multiple interacting mechanisms may be the rule rather than the exception (Jones et al. 1977). The white‐throated sparrow is a prime example. This species is polymorphic (Lowther 1961) due to a large chromosomal inversion (Huynh et al. 2011), exhibiting either a white‐stripe or tan‐stripe morph that are distinct in color and behavior (Watt et al. 1984). Nesting pairs and matings occur almost entirely between males and females from opposite morphs with same‐type pairings suffering reduced fitness, demonstrating strong disassortative mating and therefore basic rarity advantage, along with heterozygote advantage (Tuttle et al. 2016). The two morphs also exhibit consistent behavioral differences reminiscent of alternative tactics, with white‐stripe morphs exhibiting higher levels of social dominance, less parental care, and higher extra‐pair copulation. Thus, basic rarity, alternative tactics, and heterozygote advantage—both frequency‐dependent and frequency‐independent forms of maintenance selection—all may be playing a role. In another complex example, males of *Poecilia parae* guppies have five different morphs. All three types of frequency‐dependent mechanisms may be acting on the different morphs through the context of intrasexual competition for access to mates (Hurtado‐Gonzales and Uy 2009, 2010; Hurtado‐Gonzales et al. 2010).

If each type of maintenance selection can maintain phenotypic polymorphism independently, it is curious that so many polymorphisms appear to involve multiple types occurring at the same time. Above we noted that in many cases of male‐limited polymorphism, both mimicry and alternative tactics have been implicated, and that an exclusive role for overdominance appears to be rare. However, in several suspected instances of alternative tactics, overdominance also appears to be at play (e.g., Küpper et al. 2015). In addition, we noted above that heterozygote advantage is frequency‐independent but creates conditions for the evolution of disassortative mating, a form of frequency‐dependent selection. Perhaps the combination of both frequency‐dependent and frequency‐independent forms of maintenance selection can result in highly stable polymorphism. Alternatively, frequency‐independent types of maintenance selection may directly lead to frequency‐dependent forms, or vice versa, as with overdominance and disassortative mating. Does one form of maintenance selection predispose the other? Does the strength of maintenance selection increase additively when multiple types are involved? Ultimately, studying interactions may be key to understanding why some phenotypic polymorphisms persist while others do not.

### 3.2 Dimension 2: Selective Context: Ecological and Social Selection

Selection acts through the fitness of individuals, but fitness itself is multifaceted and the various processes by which natural selection occurs are numerous. Therefore, while dimension 1 describes the mechanism of trait maintenance, dimension 2 completes the hypothesis by describing the natural history context by which maintenance selection acts. Recall in our initial example comparing female polymorphism in damselflies and hummingbirds: both involve deceptive mimicry of males, but it is the selective context that differs, and this is crucial to understanding the distinction between these two hypotheses. In damselflies, *mating avoidance* is the context for male mimicry, whereas in hummingbirds, males are mimicked to *avoid aggression* and to gain access to food resources. In another comparison, damselfly and white‐throated sparrow polymorphisms, basic rarity may both play a role. However, the selective context is mate avoidance in damselflies, whereas it is mate attraction in white‐throated sparrows. To be clear, selective context is not in itself necessary to maintain polymorphic variation—this arises from maintenance selection. However, when testing alternative hypotheses for the maintenance of phenotypic polymorphism, the selective context is essential because it informs which aspect of an organism's fitness one should be observing in order to distinguish hypotheses. In other words, dimension 1 describes a process that maintains variation, whereas dimension 2 describes the aspect of fitness that is being balanced.

Although dimension 1 can be divided into a discrete number of forms, this is not so easily done with dimension 2. How can hypotheses therefore be categorized in a way that would allow for useful comparisons across taxa? Darwin (1896) recognized that there are fundamental differences between the expectations of selection involved in competition for mates (sexual selection) versus competition to survive (ecological selection). This distinction is powerful for explaining conspicuously ornamented or exaggerated traits (Andersson 1994; Hare and Simmons 2019), but it also leaves many exaggerated traits unaccounted for, such as those expressed during non‐breeding stages of an animal's life, and in females or males experiencing low levels of competition for mates (e.g., monogamous species) (West‐Eberhard 1983; Lyon and Montgomerie 2012).

The social selection framework offers a broader and more inclusive structuring of the modes of natural selection than Darwin's natural versus sexual selection framework (West‐Eberhard 1983; Lyon and Montgomerie 2012). Social selection is distinct from ecological selection, the latter of which derives from abiotic factors, prey capture ability, and interspecific interactions such as predation. In contrast, social selection arises from an individual's ability to compete with conspecifics (and sometimes heterospecifics) over any type of resource important for survival or reproduction, and views sexual section as one of several types of social selection (West‐Eberhard 1983). Darwin's sexual selection theory is therefore subsumed within the social selection framework. However, any type of competition induced by an individual's social environment may produce similar patterns as sexual selection, including weaponry or conspicuous ornamentation (West‐Eberhard 1983). Examples of non‐sexual social selection include competition for access to non‐mating breeding resources, critical food resources, or the attention of parents (West‐Eberhard 1983; Lyon and Montgomerie 2012; Tobias et al. 2012). Competition in this sense broadly encompasses both direct competition such as territorial aggression, as well as indirect competition mediated by a third party, such as mate choice, mating avoidance, or parental choice in attention toward offspring (Lyon and Montgomerie 2012). We note that while the social selection framework can be used to categorize and compare contexts, the lines between different contexts is not always clear cut, and multiple contexts may compound on each other (Wang et al. 2024). For example, access to food could have indirect implications for the ability to attract mates, or the ability to defend high‐resource territories could affect resource allocation during parental care.

While social selection provides a framework for identifying types of competition, it is not typically used as an explanation for adaptive polymorphism (but see Sinervo et al. 2001). We argue, however, that pairing social selection with maintenance selection creates a simple structure by which hypotheses for seemingly disparate types of polymorphism can be linked. Most discussion of phenotypic polymorphism focuses primarily on the influence of ecology (Clarke 1962; Endler 1978) or male competition for mates (Gross 1996; Oliveira et al. 2008). Yet, there are many other forms of competition through which maintenance selection can work to maintain polymorphism, such as mating avoidance (Fincke 2004) and access to food (Falk et al. 2022).

## 4 The Puzzle of Female‐Limited Polymorphism

We demonstrate the utility of this framework by considering female‐limited polymorphisms, where females are polymorphic but males are not. Cases of female‐limited polymorphism are sometimes considered less numerous than those in males (Oliveira et al. 2008), and occasionally are left out of discussions of sex‐limited polymorphism altogether (Gross 1996), yet female‐limited polymorphisms can be common in at least some taxa (Mank 2022). Sexual conflict and mating avoidance have been proposed as a possible context for the evolution of female‐limited polymorphism (Alonzo 2008; Svensson et al. 2009, 2025), but alternative explanations abound across taxa (e.g., Kunte 2009; Ajuria Ibarra et al. 2019; Diamant et al. 2021). It is therefore unclear whether the function of female‐limited polymorphisms can also be generalized in the same way that male‐limited polymorphisms have been (Oliveira et al. 2008; Svensson et al. 2025), and whether both female‐ and male‐limited polymorphisms can be discussed under the same conceptual framework.

We surveyed existing hypotheses for the maintenance of female‐limited polymorphisms by identifying the type of maintenance selection and the selective context (Table 1). In species where both females and males are polymorphic, we only considered cases where the polymorphism is different in males versus females (e.g., color versus size, or two versus three color morphs). Our focus was on phenotypic variation, and we did not include purely cellular, molecular, or entirely behavioral variants (see Wang et al. 2024). Polymorphisms in eusocial species were also excluded due to complex multi‐level selection in these systems, as were examples in which expression is impossible in males (e.g., egg polymorphisms). We do not seek to discredit or obviate the many hypotheses that have been suggested for female‐limited polymorphisms within specific taxa (e.g., damselflies: Fincke 1994, 2004; Andrés et al. 2002; Svensson et al. 2005; Cooper 2010; Xu and Fincke 2011; Willink and Svensson 2017). Indeed, accounting for life history is critical for developing predictions that are specific to each case of polymorphism. However, a framework for categorizing hypotheses is necessary for finding generalities, commonalities, and distinctions between different types of phenotypic polymorphism that exist in nature.

**TABLE 1:** We compiled representative species or groups of species in which the adaptive function of female‐limited polymorphisms has been studied.

			Negative frequency‐dependent mechanisms		
			Rarity advantage	Deceptive mimicry	Alternative tactics
Selective Context	Ecological	Predation avoidance		♀ Butterflies, e.g., *Papilio glaucus*	
		Prey/host detection avoidance	♀ Crab spider, *Synema globosum* ; ♀ Common cuckoo, *Cuculus canorus*	♀ Common cuckoo, *Cuculus canorus*	
	Social	Mating Avoidance	♀ Damselflies, e.g., *Ischnura* sp.; ♀ Dytiscid beetle, *Graphoderus zonatus*	♀ Damselflies, e.g., *Ischnura* sp.; ♀ African bat bug, *Afrocimex constrictus* ; ♀ Brown anole, *Anolis sagrei* ; ♀ Common cuckoo, *Cuculus canorus* ; ♀ Butterflies, e.g., *Papilio Dardanus*	
		Social Dominance	♀ Cichlid fish, *Neochromis omnicaeruleus*	♀ White‐necked jacobin, *Florisuga mellivora*	♀ Side‐blotched lizard, *Uta stansburiana*
		Competition for mates		♂	♂

*Note:* Hypotheses for polymorphism maintenance may not fall neatly into any single box, as demonstrated by examples that appear repeatedly. Most examples of female‐limited polymorphism fall into the selective context of non‐sexual social selection. We do not show frequency‐independent mechanisms here due to space, but also because there is less support for purely frequency‐independent adaptive functions for sex‐limited polymorphism. However, Schoener and Schoener (1976) proposed a heterogeneous environment hypothesis in females of *Anolis sagrei* , and a female size polymorphism in Ruffs is likely related to sexual antagonism (see Box 3 and section on heterogeneous environments) (Lank et al. 2013). Also, female *Colias* butterfly morphs may be maintained by fluctuating temperature or interference from males of another species (Nielsen and Watt 1998, 2000). Male‐limited polymorphisms have been reviewed previously, and these polymorphisms are typically attributed to alternative tactics and mimicry related to competition for mates (indicated by ♂). References included in this table: (Cook et al. 1994; Emlen 1994; Lank et al. 1995; Sinervo et al. 2000; Fincke 2004; Jukema and Piersma 2006; Reinhardt et al. 2007; Dijkstra et al. 2008; Van Gossum et al. 2008; Kunte 2009; Thorogood and Davies 2012; Karlsson Green et al. 2013; Trnka et al. 2015; Steitz et al. 2018; Ajuria Ibarra et al. 2019; Lee et al. 2019; Moon and Kamath 2019; Falk et al. 2022).

### 4.1 The Role of Social Competition

What can be learned about female‐limited polymorphism by using our proposed framework? By mapping examples onto Table 1, we see that female‐limited polymorphisms occur over a wide range of categories but are most highly concentrated under non‐sexual social competition. Others have noted that mating avoidance and sexual conflict frequently appear to underly female‐limited polymorphism, but that many other types of selection can play a role (Svensson et al. 2009, 2025; Lee et al. 2019). Our findings support this observation while also providing a broad explanatory scope that includes various forms of social selection, including mating avoidance and dominance competition. Not every case of female‐limited polymorphism, however, is driven by social interactions, as ecological contexts are also supported in some species, most notably in aposematic butterflies (reviewed in Kunte 2009). In contrast to polymorphism in females, reviews of male‐limited polymorphism have indicated a strong association with intrasexual competition for mates (Gross 1996; Taborsky 2008). Therefore, most sex‐limited polymorphisms, including both male‐ and female‐limited polymorphism, fall under the broader category of social competition (i.e., social selection), with male‐limited polymorphism primarily found more narrowly under social competition for mates (i.e., sexual selection). Competition for mates, however, has not been identified as an explanation for female‐limited polymorphism so far.

Why exactly are sex‐limited polymorphisms “limited” to a single sex? The answer may be informed in part by our hypothesis that strong social selection is a major factor in the evolution of sex‐limited polymorphism. Polymorphisms may be found in one sex because maintenance selection outweighs directional selection in one but not the other sex. For example, both males and females could in principle benefit from mimicry of another toxic species, but males might be under stronger directional sexual selection to have a non‐mimic phenotype, leading to female‐limited polymorphism. Another possibility is that a causative allele is located on a sex‐specific chromosome like the W or Y. As convenient as this might be for explaining sex‐limited traits, where associated genes have been identified they are almost always found on autosomes or on shared sex chromosomes (Svensson et al. 2025, but see Merondun et al. 2024), giving more credence to a hypothesis involving differential selection pressures on the sexes.

Another explanation that derives from the social selection framework is that competition between members of the same sex can reach very high and consistent levels over generations (West‐Eberhard 1983; Andersson 1994; Kokko and Jennions 2008; Cain and Rosvall 2014). Furthermore, especially strong social competition for mating opportunities is often found in males due to higher operational sex ratios and steeper Bateman gradients (Jennions and Kokko 2010). This may explain why male‐limited polymorphism is more common than female‐limited polymorphism (Shuster and Wade 2003; Svensson et al. 2009; Shuster 2010; Mank 2022), though Wang et al. (2024) point out that many female alternative reproductive tactics could be mostly behavioral, which we have addressed to a lesser degree here. It is interesting to note that a similar paradigm has been proposed for ornamentation and weaponry, with diverse forms of social competition driving the evolution of these phenomena in females, and strong competition for mating opportunities typically invoked to explain male‐biased expression of exaggerated traits (West‐Eberhard 1983; Tobias et al. 2012). We propose that a similar dynamic is at play in phenotypic polymorphisms: female‐limited polymorphisms are often maintained through strong social competition to increase fecundity and offspring survival, whereas male‐limited polymorphism is typically maintained through competition for access to mates. Additional testing of multiple hypotheses is necessary to confirm this hypothesis, especially because non‐mating contexts are rarely tested or considered in male‐limited polymorphisms.

Another pattern revealed in our mapping is that few examples of female‐limited polymorphism are categorized as being maintained entirely through frequency‐independent selection. Fluctuating social environments has been proposed for butterflies (Kunte 2009) and has support in at least one case in lizards, but even there, frequency‐dependent selection is likely also at play (Sinervo et al. 2000). Interestingly, reviews of male‐limited polymorphism also rarely find support for entirely frequency‐independent explanations (but see Mérot et al. 2020). This is likely because frequency‐dependent selection is inherently social and rarely exists without the interaction of individuals (Smith 1982). If social competition is a primary force in the evolution of sex‐limited polymorphism, then sex‐limited polymorphism should go hand‐in‐hand with the most socially relevant types of maintenance selection. Indeed, strong sexual selection has long been considered an evolutionary driver of polymorphism associated with alternative mating strategies in males (Gadgil 1972; Shuster 2010). Using our framework, we show that this expectation can be broadened to include polymorphism in females simply by considering competition for mates to be just one of many types of social competition for resources.

## 5 Future Questions

In addition to providing a simple method for developing alternative hypotheses, and for comparing classes of polymorphism across sexes and taxa, using this framework raises questions and avenues for future study. We briefly highlight two of these questions for further consideration.

First, how does the genetic mechanism of production of polymorphism influence the mechanisms of maintenance selection at play? Although we have focused on the adaptive maintenance of phenotypic polymorphisms, there is renewed interest in their molecular basis (Mank 2022; Kitano et al. 2025; Wellenreuther et al. 2025). For example, a number of recent studies have identified large inversion mutations as the genetic basis of several female‐limited polymorphisms (Kunte et al. 2014; Nishikawa et al. 2015; Willink et al. 2024; VanKuren et al. 2025), male‐limited polymorphisms (Küpper et al. 2015; Dodge et al. 2024; Loveland et al. 2025), and in species‐wide polymorphisms (Huynh et al. 2011; Sanchez‐Donoso et al. 2022; Kollar et al. 2025; Wellenreuther et al. 2025). In addition, other structural variants that are also associated with areas of reduced recombination, including copy number variants and transposable elements have been implicated (Willink et al. 2024; van der Bijl et al. 2025). By preventing recombination, structural variants likely create conditions for the evolution and maintenance of discrete phenotypic variation (polymorphisms) rather than continuous variation. Structural variants, however, do not always lead to balanced polymorphisms, and balanced polymorphisms do not necessarily require the prevention of recombination. It will be interesting to explore whether there are specific types of structural variants that are more likely to lead to maintenance selection, whether there are specific aspects that can predict the mechanism of maintenance selection, or whether selection is frequency‐dependent or frequency‐independent. In other words, are particular types of genetic changes more or less likely to lead to balanced polymorphism than others?

Second, how are polymorphisms that derive from cooperative interactions related to those derived from competitive interactions? We have focused primarily on phenotypic polymorphisms related to competition in males and females. Although we have ignored polymorphism in eusocial animals that may derive primarily through cooperation rather than through competition, others have noted the similarities between models of alternative tactics and cooperative breeding strategies where some individuals may focus on direct reproduction, while others forgo breeding and help their parents or siblings to breed (Koenig and Dickinson 2008). In addition, theoretical and empirical work suggests that morphs of alternative tactics contain elements of cooperation and competition (Hugie and Lank 1997; Watters 2005; Taborsky 2008; Tolliver et al. 2023). These ideas suggest an intriguing possibility that perhaps the evolution of castes in highly social insects could be unified under a common framework with alternative tactics.

## 6 Conclusion

By taking a broad view of phenotypic polymorphism, we find that both the type of maintenance selection and the context through which selection acts are two important dimensions of description for developing hypotheses to explain the adaptive maintenance of phenotypic polymorphism. By mapping instances of female‐limited polymorphism to this framework and explicitly considering the role of social selection, we show that although explanations are diverse, most proposed and supported hypotheses are associated with social competition and interaction. Ultimately, our framework aids in identifying similarities between seemingly disparate taxa, allows researchers to identify understudied topics regarding polymorphism, and clarifies distinctions between hypotheses to better understand the numerous and complex phenotypic polymorphisms found across animal taxa.

## Author Contributions

**Jay J. Falk:** conceptualization (lead), investigation (lead), visualization (lead), writing – original draft (lead), writing – review and editing (lead). **Michael S. Webster:** writing – review and editing (supporting). **Dustin R. Rubenstein:** writing – review and editing (supporting).

## Conflicts of Interest

The authors declare no conflicts of interest.

## Data Availability

No data were used in this article.

## References

[ece373493-bib-0001] Ajuria Ibarra, H. , M. Kinahan , J. Marcetteau , A. J. R. Mehigan , R. O. Ziegelmeier , and T. Reader . 2019. “The Significance of Prey Avoidance Behavior for the Maintenance of a Predator Color Polymorphism.” Behavioral Ecology 30: 240–248.

[ece373493-bib-0002] Alonzo, S. 2008. “Conflict Between the Sexes and Alternative Reproductive Tactics Within.” In Alternative Reproductive Tactics: An Integrative Approach, edited by R. F. Oliveira , M. Taborsky , and J. Brockmann , 435–450. Cambridge University Press.

[ece373493-bib-0003] Andersson, M. 1994. Sexual Selection. Princeton University Press.

[ece373493-bib-0004] Andrés, J. A. , R. A. Sánchez‐Guillén , and A. Cordero Rivera . 2002. “Evolution of Female Colour Polymorphism in Damselflies: Testing the Hypotheses.” Animal Behaviour 63: 677–685.

[ece373493-bib-0005] Askew, R. 2004. The Dragonflies of Europe. Brill.

[ece373493-bib-0006] Barrett, J. A. 1976. “The Maintenance of Non‐Mimetic Forms in a Dimorphic Batesian Mimic Species.” Evolution 30: 82–85. 28565053 10.1111/j.1558-5646.1976.tb00883.x

[ece373493-bib-0007] Bates, H. W. 1862. “XXXII. Contributions to an Insect Fauna of the Amazon Valley. Lepidoptera: Heliconidæ.” Transactions of the Linnean Society of London 23: 495–566.

[ece373493-bib-0008] Bond, A. B. 2007. “The Evolution of Color Polymorphism: Crypticity, Searching Images, and Apostatic Selection.” Annual Review of Ecology, Evolution, and Systematics 38: 489–514.

[ece373493-bib-0009] Brower, J. Z. 1960. “Experimental Studies of Mimicry. IV. The Reactions of Starlings to Different Proportions of Models and Mimics.” American Naturalist 94: 271–282.

[ece373493-bib-0010] Bull, J. J. , R. C. Vogt , and M. G. Bulmer . 1982. “Heritability of Sex Ratio in Turtles With Environmental Sex Determination.” Evolution 36: 333–341. 28563174 10.1111/j.1558-5646.1982.tb05049.x

[ece373493-bib-0011] Cain, K. E. , and K. A. Rosvall . 2014. “Next Steps for Understanding the Selective Relevance of Female‐Female Competition.” Frontiers in Ecology and Evolution 2: 32.

[ece373493-bib-0012] Canfield, M. , and E. Greene . 2009. “Phenotypic Plasticity and the Semantics of Polyphenism: A Hisorical Review and Current Perspectives.” In Phenotypic Plasticity of Insects: Mechanisms and Consequences, 65–80. Science Publishers, Inc.

[ece373493-bib-0013] Clarke, B. 1962. Balanced Polymorphism and the Diversity of Sympatric Species. Taxonomy and Geography. Systematic Association.

[ece373493-bib-0014] Clarke, B. 1964. “Frequency‐Dependent Selection for the Dominance of Rare Polymorphic Genes.” Evolution 18: 364–369.

[ece373493-bib-0015] Collins, J. P. , and J. R. Holomuzki . 1984. “Intraspecific Variation in Diet Within and Between Trophic Morphs in Larval Tiger Salamanders ( *Ambystoma tigrinum nebulosum* ).” Canadian Journal of Zoology 62: 168–174.

[ece373493-bib-0016] Connallon, T. , and A. G. Clark . 2014. “Balancing Selection in Species With Separate Sexes: Insights From Fisher's Geometric Model.” Genetics 197: 991–1006. 24812306 10.1534/genetics.114.165605 PMC4096376

[ece373493-bib-0017] Conover, D. O. , and D. A. Van Voorhees . 1990. “Evolution of a Balanced Sex Ratio by Frequency‐Dependent Selection in a Fish.” Science 250: 1556–1558. 17818284 10.1126/science.250.4987.1556

[ece373493-bib-0018] Cook, S. E. , J. G. Vernon , M. Bateson , and T. Guilford . 1994. “Mate Choice in the Polymorphic African Swallowtail Butterfly, *Papilio dardanus*: Male‐Like Females May Avoid Sexual Harassment.” Animal Behaviour 47: 389–397.

[ece373493-bib-0019] Cooper, I. A. 2010. “Ecology of Sexual Dimorphism and Clinal Variation of Coloration in a Damselfly.” American Naturalist 176: 556–572. 10.1086/656491 20860526

[ece373493-bib-0020] Cooper, I. A. , J. M. Brown , and T. Getty . 2016. “A Role for Ecology in the Evolution of Colour Variation and Sexual Dimorphism in Hawaiian Damselflies.” Journal of Evolutionary Biology 29: 418–427. 26575956 10.1111/jeb.12796

[ece373493-bib-0021] Darwin, C. 1859. On the Origin of Species by Means of Natural Selection, or the Preservation of Favoured Races in the Struggle for Life. John Murray. PMC5184128 30164232

[ece373493-bib-0022] Darwin, C. 1896. The Descent of Man and Selection in Relation to Sex. D. Appleton.

[ece373493-bib-0023] Diamant, E. S. , J. J. Falk , and D. R. Rubenstein . 2021. “Male‐Like Female Morphs in Hummingbirds: The Evolution of a Widespread Sex‐Limited Plumage Polymorphism.” Proceedings of the Royal Society B: Biological Sciences 288: 20203004. 10.1098/rspb.2020.3004 PMC7935062 33622128

[ece373493-bib-0024] Dijkstra, P. D. , O. Seehausen , and T. G. G. Groothuis . 2008. “Intrasexual Competition Among Females and the Stabilization of a Conspicuous Colour Polymorphism in a Lake Victoria Cichlid Fish.” Proceedings of the Royal Society B: Biological Sciences 275: 519–526. 10.1098/rspb.2007.1441 PMC2596810 18077255

[ece373493-bib-0025] Dobzhansky, T. 1955. A Review of Some Fundamental Concepts and Problems of Population Genetics, 1–15. Cold Spring Harbor Symposia on Quantitative Biology. Citeseer. 10.1101/sqb.1955.020.01.003 13433550

[ece373493-bib-0026] Dodge, T. O. , B. Y. Kim , J. J. Baczenas , et al. 2024. “Structural Genomic Variation and Behavioral Interactions Underpin a Balanced Sexual Mimicry Polymorphism.” Current Biology 34: 4662–4676. 39326413 10.1016/j.cub.2024.08.053 PMC12129486

[ece373493-bib-0027] Dominey, W. J. 1980. “Female Mimicry in Male Bluegill Sunfish—A Genetic Polymorphism?” Nature 284: 546–548.

[ece373493-bib-0028] Emlen, D. J. 1994. “Environmental Control of Horn Length Dimorphism in the Beetle Onthophagus Acuminatus (Coleoptera: Scarabaeidae).” Proceedings of the Royal Society of London, Series B: Biological Sciences 256: 131–136.

[ece373493-bib-0029] Endler, J. A. 1978. “A Predator's View of Animal Color Patterns.” In Evolutionary Biology, 319–364. Springer.

[ece373493-bib-0030] Falk, J. J. , C. T. Bergstrom , K. J. S. Zollman , and A. Rico‐Guevara . 2025. “Partial Honesty in a Hummingbird Polymorphism Provides Evidence for a Hybrid Equilibrium.” Animal Behaviour 222: 123104.

[ece373493-bib-0031] Falk, J. J. , D. R. Rubenstein , A. Rico‐Guevara , and M. S. Webster . 2022. “Intersexual Social Dominance Mimicry Drives Female Hummingbird Polymorphism.” Proceedings of the Royal Society B: Biological Sciences 289: 20220332. 10.1098/rspb.2022.0332 PMC9449474 36069013

[ece373493-bib-0032] Falk, J. J. , M. S. Webster , and D. R. Rubenstein . 2021. “Male‐Like Ornamentation in Female Hummingbirds Results From Social Harassment Rather Than Sexual Selection.” Current Biology 31: 4381–4387. 34450085 10.1016/j.cub.2021.07.043

[ece373493-bib-0033] Fincke, O. M. 1994. “Female Colour Polymorphism in Damselflies: Failure to Reject the Null Hypothesis.” Animal Behaviour 47: 1249–1266.

[ece373493-bib-0034] Fincke, O. M. 2004. “Polymorphic Signals of Harassed Female Odonates and the Males That Learn Them Support a Novel Frequency‐Dependent Model.” Animal Behaviour 67: 833–845.

[ece373493-bib-0035] Fisher, R. A. 1923. “XXI.—On the Dominance Ratio.” Proceedings of the Royal Society of Edinburgh 42: 321–341.

[ece373493-bib-0036] Fisher, R. A. 1930. The Genetical Theory of Natural Selection, edited by H. Bennett . Oxford University Press.

[ece373493-bib-0038] Ford, E. B. 1940. “Polymorphism and Taxonomy.” In The New Systematics, edited by J. Huxley , 493–513. Oxford University Press.

[ece373493-bib-0037] Ford, E. B. 1945. “Polymorphism.” Biological Reviews 20: 73–88.

[ece373493-bib-0039] Gadgil, M. 1972. “Male Dimorphism as a Consequence of Sexual Selection.” American Naturalist 106: 574–580.

[ece373493-bib-0040] Geffroy, B. , M. Besson , N. Sánchez‐Baizán , et al. 2021. “Unraveling the Genotype by Environment Interaction in a Thermosensitive Fish With a Polygenic Sex Determination System.” Proceedings of the National Academy of Sciences of the United States of America 118: e2112660118. 34880131 10.1073/pnas.2112660118 PMC8685686

[ece373493-bib-0041] Giraldo‐Deck, L. M. , J. L. Loveland , W. Goymann , et al. 2022. “Intralocus Conflicts Associated With a Supergene.” Nature Communications 13: 1384. 10.1038/s41467-022-29033-w PMC8927407 35296671

[ece373493-bib-0042] Graham, S. M. , W. B. Watt , and L. F. Gall . 1980. “Metabolic Resource Allocation vs. Mating Attractiveness: Adaptive Pressures on the “Alba” Polymorphism of Colias Butterflies.” Proceedings of the National Academy of Sciences 77: 3615–3619. 10.1073/pnas.77.6.3615 PMC349668 16592842

[ece373493-bib-0043] Gross, M. R. 1996. “Alternative Reproductive Strategies and Tactics: Diversity Within Sexes.” Trends in Ecology & Evolution 11: 92–98. 21237769 10.1016/0169-5347(96)81050-0

[ece373493-bib-0044] Hare, R. M. , and L. W. Simmons . 2019. “Sexual Selection and Its Evolutionary Consequences in Female Animals.” Biological Reviews 94: 929–956. 30484943 10.1111/brv.12484

[ece373493-bib-0045] Hazel, W. N. , R. Smock , and M. D. Johnson . 1990. “A Polygenic Model for the Evolution and Maintenance of Conditional Strategies.” Proceedings of the Biological Sciences 242: 181–187. 1983034 10.1098/rspb.1990.0122

[ece373493-bib-0046] Hedrick, P. W. 1986. “Genetic Polymorphism in Heterogeneous Environments: A Decade Later.” Annual Review of Ecology and Systematics 17: 535–566.

[ece373493-bib-0047] Hedrick, P. W. 1999. “Antagonistic Pleiotropy and Genetic Polymorphism: A Perspective.” Heredity 82: 126–133.

[ece373493-bib-0048] Hedrick, P. W. 2007. “Balancing Selection.” Current Biology 17: R230–R231. 17407748 10.1016/j.cub.2007.01.012

[ece373493-bib-0049] Hedrick, P. W. , M. E. Ginevan , and E. P. Ewing . 1976. “Genetic Polymorphism in Heterogeneous Environments.” Annual Review of Ecology and Systematics 7: 1–32.

[ece373493-bib-0050] Hedrick, P. W. , E. M. Tuttle , and R. A. Gonser . 2018. “Negative‐Assortative Mating in the White‐Throated Sparrow.” Journal of Heredity 109: 223–231. 29040605 10.1093/jhered/esx086 PMC6307691

[ece373493-bib-0051] Hoffman, E. A. , and D. W. Pfennig . 1999. “Proximate Causes of Cannibalistic Polyphenism in Larval Tiger Salamanders.” Ecology 80: 1076–1080.

[ece373493-bib-0052] Hughes, K. A. , A. E. Houde , A. C. Price , and F. H. Rodd . 2013. “Mating Advantage for Rare Males in Wild Guppy Populations.” Nature 503: 108–110. 24172904 10.1038/nature12717

[ece373493-bib-0053] Hugie, D. M. , and D. B. Lank . 1997. “The Resident's Dilemma: A Female Choice Model for the Evolution of Alternative Mating Strategies in Lekking Male Ruffs ( *Philomachus pugnax* ).” Behavioral Ecology 8: 218–225.

[ece373493-bib-0054] Hurtado‐Gonzales, J. L. , D. T. Baldassarre , and J. A. C. Uy . 2010. “Interaction Between Female Mating Preferences and Predation May Explain the Maintenance of Rare Males in the Pentamorphic Fish *Poecilia parae* .” Journal of Evolutionary Biology 23: 1293–1301. 20456563 10.1111/j.1420-9101.2010.01995.x

[ece373493-bib-0055] Hurtado‐Gonzales, J. L. , and J. A. C. Uy . 2009. “Alternative Mating Strategies May Favour the Persistence of a Genetically Based Colour Polymorphism in a Pentamorphic Fish.” Animal Behaviour 77: 1187–1194.

[ece373493-bib-0056] Hurtado‐Gonzales, J. L. , and J. A. C. Uy . 2010. “Intrasexual Competition Facilitates the Evolution of Alternative Mating Strategies in a Colour Polymorphic Fish.” BMC Evolutionary Biology 10: 391. 21182755 10.1186/1471-2148-10-391 PMC3017046

[ece373493-bib-0057] Huxley, J. 1942. “Evolution. The Modern Synthesis.” American Naturalist 77: 365–368.

[ece373493-bib-0059] Huynh, L. Y. , D. L. Maney , and J. W. Thomas . 2011. “Chromosome‐Wide Linkage Disequilibrium Caused by an Inversion Polymorphism in the White‐Throated Sparrow ( *Zonotrichia albicollis* ).” Heredity 106: 537–546. 20571514 10.1038/hdy.2010.85 PMC2950911

[ece373493-bib-0060] Isdaner, A. J. , N. A. Levis , I. M. Ehrenreich , and D. W. Pfennig . 2024. “Genetic Variants Underlying Plasticity in Natural Populations of Spadefoot Toads: Environmental Assessment Versus Phenotypic Response.” Genes 15: 611. 38790242 10.3390/genes15050611 PMC11121243

[ece373493-bib-0061] Jamie, G. A. 2017. “Signals, Cues and the Nature of Mimicry.” Proceedings of the Royal Society B: Biological Sciences 284: 2016–2080. 10.1098/rspb.2016.2080 PMC5326520 28202806

[ece373493-bib-0062] Jennions, M. D. , and H. Kokko . 2010. “Sexual Selection.” In Evolutionary Behavioral Ecology, edited by D. F. Westneat and C. W. Fox , 343–378. Oxford University Press.

[ece373493-bib-0063] Jones, J. S. , B. H. Leith , and P. Rawlings . 1977. “Polymorphism in Cepaea: A Problem With Too Many Solutions?” Annual Review of Ecology and Systematics 8: 109–143.

[ece373493-bib-0064] Jukema, J. , and T. Piersma . 2006. “Permanent Female Mimics in a Lekking Shorebird.” Biology Letters 2: 161–164. 17148353 10.1098/rsbl.2005.0416 PMC1618908

[ece373493-bib-0065] Kamath, A. , and A. B. Wesner . 2020. “Animal Territoriality, Property and Access: A Collaborative Exchange Between Animal Behaviour and the Social Sciences.” Animal Behaviour 164: 233–239.

[ece373493-bib-0066] Karlsson Green, K. , A. Kovalev , E. I. Svensson , and S. N. Gorb . 2013. “Male Clasping Ability, Female Polymorphism and Sexual Conflict: Fine‐Scale Elytral Morphology as a Sexually Antagonistic Adaptation in Female Diving Beetles.” Journal of the Royal Society Interface 10: 20130409. 23825114 10.1098/rsif.2013.0409 PMC3730688

[ece373493-bib-0067] Kasimatis, K. R. , T. C. Nelson , and P. C. Phillips . 2017. “Genomic Signatures of Sexual Conflict.” Journal of Heredity 108: 780–790. 29036624 10.1093/jhered/esx080 PMC5892400

[ece373493-bib-0068] Kimura, M. 1979. “The Neutral Theory of Molecular Evolution.” Scientific American 241: 98–129. 504979 10.1038/scientificamerican1179-98

[ece373493-bib-0069] Kitano, J. , K. Kagawa , T. Tsuchimatsu , R. Yamaguchi , and M. Yamamichi . 2025. “The Genomics of Discrete Polymorphisms Maintained by Disruptive Selection.” Trends in Ecology & Evolution 40: 1023–1034. 40912966 10.1016/j.tree.2025.08.003

[ece373493-bib-0070] Kocher, T. D. , R. P. Meisel , T. Gamble , K. A. Behrens , and W. J. Gammerdinger . 2024. “Yes, Polygenic Sex Determination Is a Thing!” Trends in Genetics 40: 1001–1017. 39505660 10.1016/j.tig.2024.10.003

[ece373493-bib-0071] Koenig, W. D. , and J. Dickinson . 2008. “Cooperative Breeding as an Alternative Reproductive Tactic.” In Alternative Reproductive Tactics: An Integrative Approach, edited by R. F. Oliveira , M. Taborsky , and H. J. Brockmann , 451–470. Cambridge University Press.

[ece373493-bib-0072] Kokko, H. , and M. D. Jennions . 2008. “Parental Investment, Sexual Selection and Sex Ratios.” Journal of Evolutionary Biology 21: 919–948. 18462318 10.1111/j.1420-9101.2008.01540.x

[ece373493-bib-0073] Kollar, L. M. , L. E. Stanley , S. K. Kenchanmane Raju , D. B. Lowry , and C. E. Niederhuth . 2025. “The Evolution of Locally Adaptive Chromosome Inversions in *Mimulus guttatus* .” Molecular Ecology 34: e17708. 40042128 10.1111/mec.17708 PMC12530288

[ece373493-bib-0074] Kunte, K. 2009. “Female‐Limited Mimetic Polymorphism: A Review of Theories and a Critique of Sexual Selection as Balancing Selection.” Animal Behaviour 78: 1029–1036.

[ece373493-bib-0075] Kunte, K. , W. Zhang , A. Tenger‐Trolander , et al. 2014. “Doublesex Is a Mimicry Supergene.” Nature 507: 229–232. 24598547 10.1038/nature13112

[ece373493-bib-0076] Küpper, C. , M. Stocks , J. E. Risse , et al. 2015. “A Supergene Determines Highly Divergent Male Reproductive Morphs in the Ruff.” Nature Genetics 48: 79–83. 26569125 10.1038/ng.3443 PMC5218575

[ece373493-bib-0077] Kustra, M. C. , and S. H. Alonzo . 2025. “Male Alternative Reproductive Tactics.” Current Biology 35: R697–R699. 40695232 10.1016/j.cub.2025.06.005

[ece373493-bib-0078] Lande, R. , and S. J. Arnold . 1983. “The Measurement of Selection on Correlated Characters.” Evolution 37: 1210–1226. 28556011 10.1111/j.1558-5646.1983.tb00236.x

[ece373493-bib-0079] Lank, D. B. , L. L. Farrell , T. Burke , T. Piersma , and S. B. McRae . 2013. “A Dominant Allele Controls Development into Female Mimic Male and Diminutive Female Ruffs.” Biology Letters 9: 20130653. 24196515 10.1098/rsbl.2013.0653 PMC3871350

[ece373493-bib-0080] Lank, D. B. , C. M. Smith , O. Hanotte , T. Burke , and F. Cooke . 1995. “Genetic Polymorphism for Alternative Mating Behaviour in Lekking Male Ruff *Philomachus pugnax* .” Nature 378: 59–62.

[ece373493-bib-0081] Lee, J.‐W. , H.‐N. Kim , S. Yoo , and J.‐C. Yoo . 2019. “Common Cuckoo Females May Escape Male Sexual Harassment by Color Polymorphism.” Scientific Reports 9: 7515. 31101873 10.1038/s41598-019-44024-6 PMC6525237

[ece373493-bib-0082] Levene, H. 1953. “Genetic Equilibrium When More Than One Ecological Niche Is Available.” American Naturalist 87: 331–333.

[ece373493-bib-0083] Levis, N. A. , A. J. Isdaner , and D. W. Pfennig . 2018. “Morphological Novelty Emerges From Pre‐Existing Phenotypic Plasticity.” Nature Ecology & Evolution 2: 1289–1297. 29988161 10.1038/s41559-018-0601-8

[ece373493-bib-0084] Lively, C. M. 1986. “Canalization Versus Developmental Conversion in a Spatially Variable Environment.” American Naturalist 128: 561–572.

[ece373493-bib-0085] Loveland, J. L. , A. Zemella , V. M. Jovanović , et al. 2025. “A Single Gene Orchestrates Androgen Variation Underlying Male Mating Morphs in Ruffs.” Science 387: 406–412. 39847616 10.1126/science.adp5936

[ece373493-bib-0086] Lowther, J. K. 1961. “Polymorphism in the White‐Throated Sparrow, *Zonotrichia albicollis* (Gmelin).” Canadian Journal of Zoology 39: 281–292.

[ece373493-bib-0087] Lyon, B. E. , and R. Montgomerie . 2012. “Sexual Selection Is a Form of Social Selection.” Philosophical Transactions of the Royal Society, B: Biological Sciences 367: 2266–2273. 10.1098/rstb.2012.0012 PMC3391428 22777015

[ece373493-bib-0088] Madsen, T. , B. Stille , B. Ujvari , D. Bauwens , and J. A. Endler . 2022. “Negative Frequency‐Dependent Selection on Polymorphic Color Morphs in Adders.” Current Biology 32: 3385–3388. 35714607 10.1016/j.cub.2022.05.060

[ece373493-bib-0089] Mank, J. E. 2022. “Sex‐Specific Morphs: The Genetics and Evolution of Intra‐Sexual Variation.” Nature Reviews Genetics 24: 44–52. 10.1038/s41576-022-00524-2 35971002

[ece373493-bib-0090] Mayr, E. 1963. Animal Species and Evolution. Harvard University Press.

[ece373493-bib-0091] Merondun, J. , C. I. Marques , P. Andrade , et al. 2024. “Evolution and Genetic Architecture of Sex‐Limited Polymorphism in Cuckoos.” Science Advances 10: eadl5255. 38657058 10.1126/sciadv.adl5255 PMC11042743

[ece373493-bib-0092] Mérot, C. , V. Llaurens , E. Normandeau , L. Bernatchez , and M. Wellenreuther . 2020. “Balancing Selection via Life‐History Trade‐Offs Maintains an Inversion Polymorphism in a Seaweed Fly.” Nature Communications 11: 1–11. 10.1038/s41467-020-14479-7 PMC6997199 32015341

[ece373493-bib-0093] Michener, C. D. 1961. Social Polymorphism in Hymenoptera. Insect Polymorphism 43. Royal Entomological Society.

[ece373493-bib-0094] Miller, E. T. , G. M. Leighton , B. G. Freeman , A. C. Lees , and R. A. Ligon . 2019. “Ecological and Geographical Overlap Drive Plumage Evolution and Mimicry in Woodpeckers.” Nature Communications 10: 1–10. 10.1038/s41467-019-09721-w PMC6453948 30962513

[ece373493-bib-0095] Miller, M. N. , and O. M. Fincke . 1999. “Cues for Mate Recognition and the Effect of Prior Experience on Mate Recognition in Enallagma Damselflies.” Journal of Insect Behavior 12: 801–814.

[ece373493-bib-0096] Moon, R. M. , and A. Kamath . 2019. “Re‐Examining Escape Behaviour and Habitat Use as Correlates of Dorsal Pattern Variation in Female Brown Anole Lizards, *Anolis sagrei* (Squamata: Dactyloidae).” Biological Journal of the Linnean Society 126: 783–795.

[ece373493-bib-0097] Moran, N. A. 1992. “The Evolutionary Maintenance of Alternative Phenotypes.” American Naturalist 139: 971–989.

[ece373493-bib-0098] Nielsen, M. G. , and W. B. Watt . 1998. “Behavioural Fitness Component Effects of the Alba Polymorphism of Colias (Lepidoptera, Pieridae): Resource and Time Budget Analysis.” Functional Ecology 12: 149–158.

[ece373493-bib-0099] Nielsen, M. G. , and W. B. Watt . 2000. “Interference Competition and Sexual Selection Promote Polymorphism in Colias (Lepidoptera, Pieridae).” Functional Ecology 14: 718–730.

[ece373493-bib-0100] Nijhout, H. F. 2003. “Development and Evolution of Adaptive Polyphenisms.” Evolution & Development 5: 9–18. 12492404 10.1046/j.1525-142x.2003.03003.x

[ece373493-bib-0101] Nishikawa, H. , T. Iijima , R. Kajitani , et al. 2015. “A Genetic Mechanism for Female‐Limited Batesian Mimicry in Papilio Butterfly.” Nature Genetics 47: 405–409. 25751626 10.1038/ng.3241

[ece373493-bib-0102] Oliveira, R. F. , M. Taborsky , and H. J. Brockmann . 2008. Alternative Reproductive Tactics: An Integrative Approach. Cambridge University Press.

[ece373493-bib-0103] Pfennig, D. W. , W. R. Harcombe , and K. S. Pfennig . 2001. “Frequency‐Dependent Batesian Mimicry.” Nature 410: 323. 11268195 10.1038/35066628

[ece373493-bib-0104] Plaistow, S. J. , R. A. Johnstone , N. Colegrave , and M. Spencer . 2004. “Evolution of Alternative Mating Tactics: Conditional Versus Mixed Strategies.” Behavioral Ecology 15: 534–542.

[ece373493-bib-0105] Prum, R. O. 2014. “Interspecific Social Dominance Mimicry in Birds.” Zoological Journal of the Linnean Society 172: 910–941.

[ece373493-bib-0106] Rainey, M. M. , and G. F. Grether . 2007. “Competitive Mimicry: Synthesis of a Neglected Class of Mimetic Relationships.” Ecology 88: 2440–2448. 18027745 10.1890/06-1717.1

[ece373493-bib-0107] Reinhardt, K. , E. Harney , R. Naylor , S. Gorb , and M. T. Siva‐jothy . 2007. “Female‐Limited Polymorphism in the Copulatory Organ of a Traumatically Inseminating Insect.” American Naturalist 170: 931–935. 10.1086/522844 18171174

[ece373493-bib-0108] Rice, W. R. 1992. “Sexually Antagonistic Genes: Experimental Evidence.” Science 256: 1436–1439. 10.1126/science.1604317. 1604317

[ece373493-bib-0109] Robertson, H. M. 1985. “Female Dimorphism and Mating Behaviour in a Damselfly, Ischnura Ramburi: Females Mimicking Males.” Animal Behaviour 33: 805–809.

[ece373493-bib-0110] Rosenblum, E. B. 2006. “Convergent Evolution and Divergent Selection: Lizards at the White Sands Ecotone.” American Naturalist 167: 1–15. 10.1086/498397 16475095

[ece373493-bib-0111] Ruzicka, F. , M. K. Zwoinska , D. Goedert , et al. 2025. “A Century of Theories of Balancing Selection.” Biological Reviews 101: 804–825. 41235821 10.1111/brv.70103 PMC12965862

[ece373493-bib-0112] Sanchez‐Donoso, I. , S. Ravagni , J. D. Rodríguez‐Teijeiro , et al. 2022. “Massive Genome Inversion Drives Coexistence of Divergent Morphs in Common Quails.” Current Biology 32: 462–469. 34847353 10.1016/j.cub.2021.11.019

[ece373493-bib-0113] Schoener, T. W. , and A. Schoener . 1976. “The Ecological Context of Female Pattern Polymorphism in the Lizard *Anolis sagrei* .” Evolution 30: 650–658. 28563330 10.1111/j.1558-5646.1976.tb00946.x

[ece373493-bib-0114] Shine, R. , G. P. Brown , and C. Goiran . 2022. “Frequency‐Dependent Batesian Mimicry Maintains Colour Polymorphism in a Sea Snake Population.” Scientific Reports 12: 4680. 35304528 10.1038/s41598-022-08639-6 PMC8933499

[ece373493-bib-0115] Shuster, S. M. 1992. “The Reproductive Behaviour of α‐, β‐, and γ‐Male Morphs in *Paracerceis sculpta* , a Marine Isopod Crustacean.” Behaviour 121: 231–257.

[ece373493-bib-0116] Shuster, S. M. 2010. “Alternative Mating Strategies.” In Evolutionary Behavioral Ecology, edited by D. F. Westneat and C. W. Fox , 434–450. Oxford University Press.

[ece373493-bib-0117] Shuster, S. M. , and M. J. Wade . 1991. “Equal Mating Success Among Male Reproductive Strategies in a Marine Isopod.” Nature 350: 608–610.

[ece373493-bib-0118] Shuster, S. M. , and M. J. Wade . 2003. Mating Systems and Strategies. Princeton University Press.

[ece373493-bib-0119] Simmons, L. W. , and D. J. Emlen . 2006. “Evolutionary Trade‐Off Between Weapons and Testes.” Proceedings of the National Academy of Sciences 103: 16346–16351. 10.1073/pnas.0603474103 PMC1637585 17053078

[ece373493-bib-0120] Sinervo, B. , C. Bleay , and C. Adamopoulou . 2001. “Social Causes of Correlational Selection and the Resolution of a Heritable Throat Color Polymorphism in a Lizard.” Evolution 55: 2040–2052. 11761064 10.1111/j.0014-3820.2001.tb01320.x

[ece373493-bib-0121] Sinervo, B. , E. Svensson , and T. Comendant . 2000. “Density Cycles and an Offspring Quantity and Quality Game Driven by Natural Selection.” Nature 406: 985–988. 10984050 10.1038/35023149

[ece373493-bib-0122] Smith, J. M. 1982. Evolution and the Theory of Games. Cambridge university press.

[ece373493-bib-0123] Steitz, I. , C. Kingwell , R. J. Paxton , and M. Ayasse . 2018. “Evolution of Caste‐Specific Chemical Profiles in Halictid Bees.” Journal of Chemical Ecology 44: 827–837. 30014321 10.1007/s10886-018-0991-8

[ece373493-bib-0124] Strickland, L. R. , C. F. Arias , V. Rodriguez , J. S. Johnston , W. O. McMillan , and D. Windsor . 2019. “Inheritance, Distribution and Genetic Differentiation of a Color Polymorphism in Panamanian Populations of the Tortoise Beetle, *Chelymorpha alternans* (Coleoptera: Chrysomelidae).” Heredity 122: 558–569. 30315219 10.1038/s41437-018-0149-z PMC6462003

[ece373493-bib-0125] Strickland, L. R. , R. C. Fuller , D. Windsor , and C. E. Cáceres . 2021. “A Potential Role for Overdominance in the Maintenance of Colour Variation in the Neotropical Tortoise Beetle, *Chelymorpha alternans* .” Journal of Evolutionary Biology 34: 779–791. 33704867 10.1111/jeb.13779

[ece373493-bib-0126] Svensson, E. I. 2023. “Phenotypic Selection in Natural Populations: What Have We Learned in 40 Years?” Evolution 77: 1493–1504. 37105948 10.1093/evolut/qpad077

[ece373493-bib-0127] Svensson, E. I. , J. Abbott , and R. Härdling . 2005. “Female Polymorphism, Frequency Dependence, and Rapid Evolutionary Dynamics in Natural Populations.” American Naturalist 165: 567–576. 10.1086/429278 15795853

[ece373493-bib-0128] Svensson, E. I. , J. K. Abbott , T. P. Gosden , and A. Coreau . 2009. “Female Polymorphisms, Sexual Conflict and Limits to Speciation Processes in Animals.” Evolutionary Ecology 23: 93–108.

[ece373493-bib-0129] Svensson, E. I. , J. Falk , L. Iversen , et al. 2025. “The Origin, Genomics and Evolution of Female‐Limited Polymorphisms.” Philosophical Transactions of the Royal Society, B: Biological Sciences. In press. 10.1098/rstb.2025.0175 42272386

[ece373493-bib-0130] Taborsky, M. 2008. “Alternative Reproductive Tactics in Fish.” In Alternative Reproductive Tactics an Integrative Approach, edited by R. F. Oliveira , M. Taborsky , and H. J. Brockmann . Cambridge University Press.

[ece373493-bib-0131] Thorogood, R. , and N. B. Davies . 2012. “Cuckoos Combat Socially Transmitted Defenses of Reed Warbler Hosts With a Plumage Polymorphism.” Science 337: 578–580. 22859487 10.1126/science.1220759

[ece373493-bib-0132] Tobias, J. A. , R. Montgomerie , and B. E. Lyon . 2012. “The Evolution of Female Ornaments and Weaponry: Social Selection, Sexual Selection and Ecological Competition.” Philosophical Transactions of the Royal Society, B: Biological Sciences 367: 2274–2293. 10.1098/rstb.2011.0280 PMC3391421 22777016

[ece373493-bib-0133] Tolliver, J. D. M. , K. Kupán , D. B. Lank , S. Schindler , and C. Küpper . 2023. “Fitness Benefits From Co‐Display Favour Subdominant Male–Male Partnerships Between Phenotypes.” Animal Behaviour 197: 131–154.

[ece373493-bib-0134] Tomkins, J. L. , and W. Hazel . 2007. “The Status of the Conditional Evolutionarily Stable Strategy.” Trends in Ecology & Evolution 22: 522–528. 17919770 10.1016/j.tree.2007.09.002

[ece373493-bib-0135] Trnka, A. , M. Trnka , and T. Grim . 2015. “Do Rufous Common Cuckoo Females Indeed Mimic a Predator? An Experimental Test.” Biological Journal of the Linnean Society 116: 134–143.

[ece373493-bib-0136] Tsai, H.‐Y. , D. R. Rubenstein , Y.‐M. Fan , et al. 2020. “Locally‐Adapted Reproductive Photoperiodism Determines Population Vulnerability to Climate Change in Burying Beetles.” Nature Communications 11: 1398. 10.1038/s41467-020-15208-w PMC7069978 32170152

[ece373493-bib-0137] Tuttle, E. M. , A. O. Bergland , M. L. Korody , et al. 2016. “Divergence and Functional Degradation of a Sex Chromosome‐Like Supergene.” Current Biology 26: 344–350. 26804558 10.1016/j.cub.2015.11.069 PMC4747794

[ece373493-bib-0138] Twyford, A. D. , and J. Friedman . 2015. “Adaptive Divergence in the Monkey Flower *Mimulus guttatus* Is Maintained by a Chromosomal Inversion.” Evolution 69: 1476–1486. 25879251 10.1111/evo.12663 PMC5029580

[ece373493-bib-0139] van der Bijl, W. , J. J. Shu , V. S. Goberdhan , et al. 2025. “Deep Learning Reveals the Complex Genetic Architecture of Male Guppy Colouration.” Nature Ecology & Evolution 9: 1614–1625. 40596731 10.1038/s41559-025-02781-w

[ece373493-bib-0140] Van Gossum, H. , T. N. Sherratt , A. Cordero‐Rivera , and A. Córdoba‐Aguilar . 2008. “The Evolution of Sex‐Limited Colour Polymorphism.” In Dragonflies and Damselflies: Model Organisms for Ecological and Evolutionary Research, edited by A. Córdoba‐Aguilar , 219–231. Oxford University Press.

[ece373493-bib-0141] VanKuren, N. W. , S. I. Sheikh , C. L. Fu , et al. 2025. “Functional Genetic Elements of a Butterfly Mimicry Supergene.” Proceedings of the National Academy of Sciences of the United States of America 122: e2509864122. 41060750 10.1073/pnas.2509864122 PMC12541413

[ece373493-bib-0142] Verhaar, H. 1985. “Guide des Libellules D'Europe et D'Afrique du Nord.” Contactblad Nederlandse Libellenonderzoekers 10: 18–19.

[ece373493-bib-0143] Wallace, A. R. 1865. “I. On the Phenomena of Variation and Geographical Distribution as Illustrated by the Papilionidæ of the Malayan Region.” Transactions of the Linnean Society of London 1: 1–71.

[ece373493-bib-0144] Wallace, B. 1975. “Hard and Soft Selection Revisited.” Evolution 29: 465–473. 28563194 10.1111/j.1558-5646.1975.tb00836.x

[ece373493-bib-0145] Wang, D. , J. Abbott , F. A. Brenninger , et al. 2024. “Female Alternative Reproductive Tactics: Diversity and Drivers.” Trends in Ecology & Evolution 39: 937–946. 38955568 10.1016/j.tree.2024.06.002

[ece373493-bib-0146] Watt, D. J. , C. J. Ralph , and C. T. Atkinson . 1984. “The Role of Plumage Polymorphism in Dominance Relationships of the White‐Throated Sparrow.” Auk 101: 110–120.

[ece373493-bib-0147] Watters, J. V. 2005. “Can the Alternative Male Tactics ‘Fighter’ and ‘Sneaker’ Be Considered ‘Coercer’ and ‘Cooperator’ in Coho Salmon?” Animal Behaviour 70: 1055–1062.

[ece373493-bib-0148] Wellenreuther, M. , R. A. Oomen , K. Y. Han , R. Krohman , and T. B. H. Reusch . 2025. “Beyond Supergenes: The Diverse Roles of Inversions in Trait Evolution.” Trends in Ecology & Evolution 40: 1054–1065. 40912967 10.1016/j.tree.2025.08.004

[ece373493-bib-0149] West‐Eberhard, M. J. 1983. “Sexual Selection, Social Competition, and Speciation.” Quarterly Review of Biology 58: 155–183.

[ece373493-bib-0150] West‐Eberhard, M. J. 2003a. “Cross‐Sexual Transfer.” In Developmental Plasticity and Evolution, 260–295. Oxford University Press.

[ece373493-bib-0151] West‐Eberhard, M. J. 2003b. Developmental Plasticity and Evolution. Oxford University Press.

[ece373493-bib-0152] Willink, B. , T. A. T. Ho , and E. I. Svensson . 2025. “Ecology and Sexual Conflict Drive the Macroevolutionary Dynamics of Female‐Limited Colour Polymorphisms.” Evolutionary Biology 122: e2503400122. 10.1073/pnas.2503400122 PMC12435309 40906803

[ece373493-bib-0153] Willink, B. , and E. I. Svensson . 2017. “Intra‐ and Intersexual Differences in Parasite Resistance and Female Fitness Tolerance in a Polymorphic Insect.” Proceedings of the Royal Society B: Biological Sciences 284: 20162407. 10.1098/rspb.2016.2407 PMC5310041 28123090

[ece373493-bib-0154] Willink, B. , K. Tunström , S. Nilén , et al. 2024. “The Genomics and Evolution of Inter‐Sexual Mimicry and Female‐Limited Polymorphisms in Damselflies.” Nature Ecology & Evolution 8: 83–97. 37932383 10.1038/s41559-023-02243-1 PMC10781644

[ece373493-bib-0155] Xu, M. , and O. M. Fincke . 2011. “Tests of the Harassment‐Reduction Function and Frequency‐Dependent Maintenance of a Female‐Specific Color Polymorphism in a Damselfly.” Behavioral Ecology and Sociobiology 65: 1215–1227.

